# A Theoretical and Empirical Integrated Method to Select the Optimal Combined Signals for Geometry-Free and Geometry-Based Three-Carrier Ambiguity Resolution

**DOI:** 10.3390/s16111929

**Published:** 2016-11-16

**Authors:** Dongsheng Zhao, Gethin Wyn Roberts, Lawrence Lau, Craig M. Hancock, Ruibin Bai

**Affiliations:** 1 International Doctoral Innovation Center, The University of Nottingham Ningbo China, 199 Taikang East Road, Ningbo 315100, China; 2 Department of Civil Engineering, The University of Nottingham Ningbo China, 199 Taikang East Road, Ningbo 315100, China; Lawrence.Lau@nottingham.edu.cn (L.L.); Craig.Hancock@nottingham.edu.cn (C.M.H.); 3 Nottingham Geospatial Institute, The University of Nottingham, Triumph Road, Nottingham NG72TU, UK; 4 School of Computer Science, The University of Nottingham Ningbo China, 199 Taikang East Road, Ningbo 315100, China; Ruibin.Bai@nottingham.edu.cn

**Keywords:** GPS, ambiguity resolution, geometry-free, geometry-based, triple-frequency observations, real data

## Abstract

Twelve GPS Block IIF satellites, out of the current constellation, can transmit on three-frequency signals (L1, L2, L5). Taking advantages of these signals, Three-Carrier Ambiguity Resolution (TCAR) is expected to bring much benefit for ambiguity resolution. One of the research areas is to find the optimal combined signals for a better ambiguity resolution in geometry-free (GF) and geometry-based (GB) mode. However, the existing researches select the signals through either pure theoretical analysis or testing with simulated data, which might be biased as the real observation condition could be different from theoretical prediction or simulation. In this paper, we propose a theoretical and empirical integrated method, which first selects the possible optimal combined signals in theory and then refines these signals with real triple-frequency GPS data, observed at eleven baselines of different lengths. An interpolation technique is also adopted in order to show changes of the AR performance with the increase in baseline length. The results show that the AR success rate can be improved by 3% in GF mode and 8% in GB mode at certain intervals of the baseline length. Therefore, the TCAR can perform better by adopting the combined signals proposed in this paper when the baseline meets the length condition.

## 1. Introduction

At the time of writing, three-frequency signals are available from all 12 GPS Block IIF satellites. One of the new generation of GPS satellites, GPS III or GPS Block IIIA, is planned to be initially launched on 3 May 2017, bringing another signal, L1C. The additional signals are anticipated to bring significant improvements to the efficiency and reliabilities of carrier phase ambiguity resolution (AR) by forming more extra-wide-lane (EWL) and wide-lane (WL) combinations. The selection of optimal signal combinations has become a popular topic since optimal signal combinations can not only eliminate or mitigate bias terms in the mathematical model but also alleviate the computational burden of processing multi-frequency data [1]. Therefore, significant research has been conducted towards carrier phase AR using triple-frequency signals during the past decade. The idea of triple-frequency cascading AR was initially studied by Forssell et al. [2] and Vollath et al. [3], who described the Three-Carrier Ambiguity Resolution (TCAR) method for GNSS-2. De Jonge et al. [4] and Hatch et al. [5] proposed the cascaded integer resolution (CIR) method for GPS. This method was expanded to multi-carrier signals by Werner and Winkel [6] and Ji et al. [7]. All these classical methods are essentially geometry-free (GF) methods, in which the geometric distance, orbit error, and tropospheric bias are eliminated, leaving only the residuals of the ionospheric biases. Therefore, the early TCAR method can only be applied to the short baseline due to ionospheric biases [8]. The ionospheric biases can be considered as systematic errors, whose characteristics were systematically analyzed by Sieradzki and Paziewski [9,10], Zhang et al. [11]. In order to reduce the effect of the ionospheric bias, Feng [12] formed the ionosphere-reduced signal combinations in order to enhance the conventional TCAR and expand the application range. Li et al. [13] made a further improvement by proposing a GF and ionosphere-free model to fix the integer ambiguities using just several minutes of data and without the limitation of distance. However, their method was only tested using semi-generated triple-frequency GPS signals. The same method was tested using real triple-frequency GPS and Galileo data by Wang and Rothacher [14], who suggested that the improvement factor would be better when more triple-frequency satellites are available. The ionospheric bias can also be eliminated or reduced by employing the ambiguity resolved EWL combination and three pseudorange observations in the second step of TCAR [15,16].

Although early TCAR can only resolve integer ambiguities using a GF bootstrapping methods [17], it has been proved that TCAR can also be implemented in a geometry-based (GB) process. Feng and Li [18,19] presented a general linear equation system for a GB TCAR model, whose performance was analyzed in [20] using semi-generated GPS triple-frequency signals with a result that the success rate was much better than the dual-frequency case. Tang et al. [21] improved the GB TCAR model with the estimation of the slant ionosphere bias at each epoch, resulting in high AR success rate in short baseline applications using real data of BeiDou System (BDS). He et al. [22] presented a WL real-time positioning algorithm based on BDS triple-frequency signals. Gao et al. [23] proposed a BDS and GPS combined GB AR model testing with triple-frequency BDS data and dual-frequency GPS data. Besides using the traditional bootstrapping method, another ambiguity search method is the Least-square Ambiguity Decorrelation Adjustment (LAMBDA) search method, for which AR performance was proved to be better than bootstrapping by Teunissen [17]. The research of applying the LAMBDA method to the multi-frequency AR has been conducted by a number of papers [24,25,26,27,28]. Therefore, in this study, the LAMBDA method is adopted as the ambiguity search method for combined GPS signals.

The purpose of this study is to identify the optimal signal combinations for GF and GB TCAR separately with regards to different baseline lengths, which usually mean different levels of biases including both ionospheric biases and tropospheric biases. This study is significant for the following two reasons. Most of the research described above selected only one optimal signal combination with the longest wavelength, the lowest noise, the lowest total noise level or the lowest ionospheric scale factor (ISF) for each step of AR, expecting this combination could work well in all conditions [12,18]. However, this is not the case when the length of the baseline is changed. Therefore, in this study, besides considering the wavelength, noise and ISF, we will choose the optimal combinations with regards to the effect of different levels of biases, caused by the changes of baseline lengths, instead of the natural phenomenon such as scintillation. In addition, this research is of importance because real triple-frequency GPS data from eleven baselines with different lengths are adopted to refine the theoretically selected combined signals while the signals proposed by most of the research above were tested with simulated data. The benefit of using the real data is that the selected signals are more representative for real field surveying. These signals can also help engineers design location applications with higher positioning performance by providing more solid ambiguities. Although Wang and Rothacher [14] adopted real GPS data, at that time there were only two triple-frequency GPS satellites in orbit, which were not enough for deciding GB ambiguities and positioning. In this study, one hour of real GPS data with 6 triple-frequency satellites available was collected to test the optimal signals for both GF TCAR and GB TCAR separately.

This paper is organized as follows. In Section 2, after a brief introduction to both the GF TCAR model and the GB model, the optimal signal combinations will be selected theoretically for both models separately, followed by the analysis of these combinations. In Section 3, the selected combinations will be corroborated by testing with real GPS data followed by the analysis of the results, in relation to the different levels of ionospheric biases. Finally in the last section, conclusions will be drawn.

## 2. Theoretical Experiment and Result Analysis

### 2.1. Basic Concept of GF and GB TCAR Using Combined Signals

Generally, both GF and GB TCAR methods adopted in this study are based on a group of selected linear combinations of the multi-frequency observations. A general form of GPS triple-frequency signal combinations for double-difference (DD) carrier phase $\nabla ▵\Phi_{\left(i,j,k\right)}$ and DD pseudorange $\nabla ▵P_{\left(i,j,k\right)}$ are defined as follows [12].

(1) $$\nabla ▵\Phi_{\left(i,j,k\right)}=\frac{i\cdot f_{1}\cdot \nabla ▵\Phi_{1}+j\cdot f_{2}\cdot \nabla ▵\Phi_{2}+k\cdot f_{5}\cdot \nabla ▵\Phi_{5}}{i\cdot f_{1}+j\cdot f_{2}+k\cdot f_{5}}$$

(2) $$\nabla ▵P_{\left(i,j,k\right)}=\frac{i\cdot f_{1}\cdot \nabla ▵P_{1}+j\cdot f_{2}\cdot \nabla ▵P_{2}+k\cdot f_{5}\cdot \nabla ▵P_{5}}{i\cdot f_{1}+j\cdot f_{2}+k\cdot f_{5}}$$

where *i*, *j* and *k* are the coefficients, which must be integer numbers in order to maintain the integral property of the ambiguity; $f_{1}$, $f_{2}$ and $f_{5}$ are the frequencies of the carrier phase measurements on signals L1, L2 and L5 in units of Hz; $\nabla ▵\Phi_{1}$, $\nabla ▵\Phi_{2}$ and $\nabla ▵\Phi_{5}$ represent L1, L2 and L5 DD carrier phase observations respectively for GPS in units of length; $\nabla ▵P_{1}$, $\nabla ▵P_{2}$ and $\nabla ▵P_{5}$ represent L1, L2 and L5 DD pseudorange observations respectively for GPS in units of length. The corresponding frequency $f_{\left(i,j,k\right)}$, wavelength $\lambda_{\left(i,j,k\right)}$ and integer ambiguity of the DD combined carrier phase measurement $\nabla ▵N_{\left(i,j,k\right)}$ are defined as follows.

(3) $$f_{\left(i,j,k\right)}=i\cdot f_{1}+j\cdot f_{2}+k\cdot f_{5}$$

(4) $$\lambda_{\left(i,j,k\right)}=\frac{c}{i\cdot f_{1}+j\cdot f_{2}+k\cdot f_{5}}$$

(5) $$\nabla ▵N_{\left(i,j,k\right)}=i\cdot \nabla ▵N_{1}+j\cdot \nabla ▵N_{2}+k\cdot \nabla ▵N_{5}$$

where *c* is the speed of light in vacuum; $\nabla ▵N_{1}$, $\nabla ▵N_{2}$ and $\nabla ▵N_{5}$ are the L1, L2 and L5 DD ambiguities of the carrier phase measurements in units of cycles. With regards to the general GPS observation equation [29], the observation equations of DD carrier phase and DD pseudorange on the combined signals can be denoted as follows.

(6) $$\nabla ▵\Phi_{\left(i,j,k\right)}=\nabla ▵\rho +\nabla ▵\delta_{orb}+\nabla ▵\delta_{trop}-\beta_{\left(i,j,k\right)}\nabla ▵I_{1}-\lambda_{\left(i,j,k\right)}\nabla ▵N_{\left(i,j,k\right)}+\varepsilon_{\nabla ▵\Phi_{\left(i,j,k\right)}}$$

(7) $$\nabla ▵P_{\left(i,j,k\right)}=\nabla ▵\rho +\nabla ▵\delta_{orb}+\nabla ▵\delta_{trop}+\beta_{\left(i,j,k\right)}\nabla ▵I_{1}+\varepsilon_{\nabla ▵P_{\left(i,j,k\right)}}$$

where ∇▵ρ represents the DD geometric distance from satellites to receivers; $\nabla ▵\delta_{orb}$ is the DD satellite orbit error; $\nabla ▵\delta_{trop}$ is the DD tropospheric delay; $\nabla ▵I_{1}$ is the first-order ionospheric bias on GPS L1 carrier; *ε* represents the observation noise; $\beta_{\left(i,j,k\right)}$is known as the ionospheric scale factor (ISF). It indicates the ionospheric influence level on the linear combination compared to that on carrier L1, as well as the difficulty for AR using this linear combination. The phase noise factor (PNF) $\mu_{\left(i,j,k\right)}^{2}$ is used to denote the noise level of the combined phase and combined code measurements [12,23]. $\beta_{\left(i,j,k\right)}$ and $\mu_{\left(i,j,k\right)}^{2}$ can be expressed as follows.

(8) $$\beta_{\left(i,j,k\right)}=\frac{f_{1}^{2}\left(i/f_{1}+j/f_{2}+k/f_{5}\right)}{i\cdot f_{1}+j\cdot f_{2}+k\cdot f_{5}}$$

(9) $$\mu_{\left(i,j,k\right)}^{2}=\frac{{\left(i\cdot f_{1}\right)}^{2}+{\left(j\cdot f_{2}\right)}^{2}+{\left(k\cdot f_{5}\right)}^{2}}{{\left(i\cdot f_{1}+j\cdot f_{2}+k\cdot f_{5}\right)}^{2}}$$

The general mathematical model for GF TCAR is outlined in the following steps. The details of GF TCAR can be found in [30].

- Step 1. Fixing the EWL ambiguities.
  (10) $$\nabla ▵N_{\text{EWL}}=\frac{1}{\lambda_{\text{EWL}}}\left[\nabla ▵P_{\text{code}}-\nabla ▵\Phi_{\text{EWL}}-\left(\beta_{\text{code}}+\beta_{\text{EWL}}\right)\nabla ▵I_{1}\right]$$
  (11) $$\nabla ▵{\hat{N}}_{\text{EWL}}={\left[\nabla ▵N_{\text{EWL}}\right]}_{\text{round-off}}$$
  (12) $$\nabla ▵{\overset{ˇ}{\Phi }}_{\text{EWL}}=\nabla ▵\Phi_{\text{EWL}}+\lambda_{\text{EWL}}\nabla ▵{\overset{ˇ}{N}}_{\text{EWL}}$$
- Step 2. Fixing WL ambiguities.
  (13) $$\nabla ▵N_{\text{WL}}=\frac{1}{\lambda_{\text{WL}}}\left[\nabla ▵{\overset{ˇ}{\Phi }}_{\text{EWL}}-\nabla ▵\Phi_{\text{WL}}+\left(\beta_{\text{WL}}-\beta_{\text{EWL}}\right)\nabla ▵I_{1}\right]$$
  (14) $$\nabla ▵{\overset{ˇ}{N}}_{\text{WL}}={\left[\nabla ▵N_{\text{WL}}\right]}_{\text{round-off}}$$
  (15) $$\nabla ▵{\overset{ˇ}{\Phi }}_{\text{WL}}=\nabla ▵\Phi_{\text{WL}}+\lambda_{\text{WL}}\nabla ▵{\overset{ˇ}{N}}_{\text{WL}}$$
- Step 3. Fixing the ambiguities of fundamental signals.
  (16) $$\nabla ▵N_{\text{single}}=\frac{1}{\lambda_{\text{single}}}\left[\nabla ▵{\overset{ˇ}{\Phi }}_{\text{WL}}-\nabla ▵\Phi_{\text{single}}+\left(\beta_{\text{single}}-\beta_{\text{WL}}\right)\nabla ▵I_{1}\right]$$
  (17) $$\nabla ▵{\overset{ˇ}{N}}_{\text{single}}={\left[\nabla ▵N_{\text{single}}\right]}_{\text{round-off}}$$
  (18) $$\nabla ▵{\overset{ˇ}{\Phi }}_{\text{single}}=\nabla ▵\Phi_{\text{single}}+\lambda_{\text{single}}\nabla ▵{\overset{ˇ}{N}}_{\text{single}}$$

where code means pseudorange measurements; EWL is the extra-wide-lane carrier phase measurement (λ≥2.93 m); WL is the wide-lane carrier phase measurements (0.75 m ≤λ<2.93 m); single means the fundamental signal, denotes the ambiguity corrected observation. The ambiguities and the ambiguity corrected phase measurements are the parameters that need to be estimated. The traditional signal group for GF TCAR includes EWL(0, 1, −1), WL(1, −1, 0) and single(1, 0, 0) [30].

The general mathematical model for GB TCAR is outlined in the following steps. The details of GB TCAR can be found in [20,31,32]

- Step 1. Fixing the EWL ambiguities in a GF model. In this step, instead of using a GB model, a GF model (Equations (10)–(12)) is usually adopted, as there is no difference in the success rate between them and it is more convenient to conduct AR in a GF model [11].
- Step 2. Fixing the ambiguities of the second EWL/WL signals.
  (19) $$\left[\begin{array}{c} \nabla ▵{\overset{ˇ}{\Phi }}_{\text{EWL}}-\nabla ▵\rho_{0}\\ \nabla ▵\Phi_{W2}-\nabla ▵\rho_{0} \end{array}\right]=\left[\begin{array}{cc} A & 0\\ A & -I\cdot \lambda_{W2} \end{array}\right]\left[\begin{array}{c} \delta x\\ N_{W2} \end{array}\right]+\left[\begin{array}{c} \varepsilon_{\nabla ▵P}\\ \varepsilon_{\nabla ▵\Phi_{W2}} \end{array}\right]$$
- Step 3. Fixing the ambiguities of the NL signal.
  (20) $$\left[\begin{array}{c} \nabla ▵{\overset{ˇ}{\Phi }}_{W2}-\nabla ▵\rho_{0}\\ \nabla ▵\Phi_{W3}-\nabla ▵\rho_{0} \end{array}\right]=\left[\begin{array}{cc} A & 0\\ A & -I\cdot \lambda_{W3} \end{array}\right]\left[\begin{array}{c} \delta x\\ N_{W3} \end{array}\right]+\left[\begin{array}{c} \varepsilon_{\nabla ▵P}\\ \varepsilon_{\nabla ▵\Phi_{W3}} \end{array}\right]$$

where *A* is the linear coefficient matrix; δx is the baseline parameter; W2, W3 denote the signal combinations used in the GB TCAR model. The original ambiguities of the three fundamental signals can be recovered from the three ambiguity resolved independent combined signals. The conventional signal group for GB TCAR includes EWL(0, 1, −1), EWL(1, −6, 5) and NL(4, 0, −3) [12]. Equations (19) and (20) can be denoted as the following formart.

(21) L=BX+V

where *L* is the observation vector; *B* is the coefficient matrix; *X* is the unknown parameter vector including both the baseline parameters and the ambiguities, which need to be estimated; *V* is the residual vector. Equation (21) can be solved by least-squares (LSQ) adjustment, where the LAMBDA search method can be used to calculate the fixed solutions. With regards of the correlation of DD measurements and the correlation of the two combined signals, the variance-covariance matrix ($Q_{L}$) of the observation vector is denoted in Equation (22), where (i,j,k) and (a,b,c) represent different signal combinations. The weight matrix (*P*) and the stochastic model of the estimated parameters ($Q_{X}$) are denoted in Equations (23) and (24) respectively.

(22) $$Q_{L}=\left[\begin{array}{cc} Q_{\nabla ▵\Phi_{\left(i,j,k\right)}} & Q_{\nabla ▵\Phi_{\left(i,j,k\right)},\nabla ▵\Phi_{\left(a,b,c\right)}}\\ Q_{\nabla ▵\Phi_{\left(a,b,c\right)},\nabla ▵\Phi_{\left(i,j,k\right)}} & Q_{\nabla ▵\Phi_{\left(a,b,c\right)}} \end{array}\right]$$

(23) $$P=Q_{L}^{-1}$$

(24) $$Q_{X}={\left(B^{T}PB\right)}^{-1}$$

### 2.2. Selection of Combined Signals Based on Theoretical Consultation and Result Analysis

#### 2.2.1. Theoretical Selection of Combined Signals for GF TCAR

**Figure 1:**
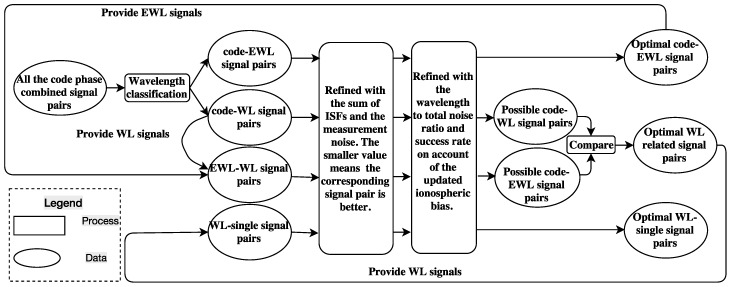
The procedure of selecting the optimal signal pairs for GF TCAR.

GF TCAR can eliminate most of the effect of the error sources, such as the satellite orbit error and the tropospheric bias, by adopting a pair of combined signals in each step. Taking advantage of the triple-frequency signals, the optimal combined EWL and WL signals can significantly enhance the efficiency and reliability of the AR [33,34]. However, many researchers proposed combined signals often only considered the EWL or WL signal independently instead of the signal pair [12,16,21]. Therefore, the main aim of this subsection is to discover the optimal combined signal pair in theory to fix the ambiguities more easily, mainly considering the following four criteria, wavelength, the sum of ISFs and the measurement noise, the wavelength to total noise ratio and the theoretical success rate. Figure 1 summarizes the procedure of selecting the optimal signal pairs for GF TCAR using these four criteria.

As combined signal pairs of different wavelengths are needed in different steps of GF TCAR, we will select two groups of combined signal pairs, EWL or WL, whose ambiguities are to be determined. The numerous selected signal pairs will be refined with the sum of ISFs and the measurement noise ($\sigma_{\mathrm{GF}}$), which is calculated by Equation (25) and denoted in units of cycles. Usually, a smaller value of the sum of the ISFs means the corresponding signal pair is less affected by the ionospheric bias, while a smaller measurement noise means more precision of the signal pair. Both of them contribute to AR. Ideally, signal pairs with the smallest sum of ISFs and measurement noise at the same time should be pursed, which is not often the case. In general, the ionospheric bias contributes the most error in the long baseline, while in the zero or short baseline effects of the measurement noise or the multipath are more considerable respectively. Thus the combined signals obtained based on the smallest ISF only may not be suitable for the zero or short baseline. It is a complex work to model the effect of the multipath, which is not considered to be a common factor when deciding the signals for AR as well. Thus, only the sum of ISFs and the measurement noise are taken into account in order to find the suitable signal combination pairs for different levels of ionospheric bias. After considering the ISF and the measurement noise, there will be still too many options left, which can be further refined with the wavelength to total noise ratio (ratio) and the success rate considering the updated ionospheric bias. The optimal signal pairs will be those with the largest wavelength to total noise ratio and the largest success rate. In this study, the measurement noise of the DD carrier phase on L1 is set to 0.5 cm, while the DD code noise is 50 cm [13]. The ionospheric biases are linearly increased from 0 to 100 cm with the increase of the baseline length from 0 to 500 km [13]. The total noise of the combined signal pair can be calculated by Equation (26). It should be noted that the selection of the mathematical symbol depends on whether the signal pair is a code and phase mixed pair or a pure phase pair. When a pure phase pair is adopted, the mathematical symbol is—in Equation (26), while $\sigma_{\nabla ▵P_{1}}$ should be replaced with $\sigma_{\nabla ▵\Phi_{1}}$ in both Equations (25) and (26). The combined signal with coefficients (i,j,k) is the one with ambiguities to be determined. Equation (27) is for the calculation of the wavelength to total noise ratio.

(25) $$\sigma_{\mathrm{GF}}=\frac{1}{\lambda_{\left(i,j,k\right)}}\sqrt{\mu_{\left(a,b,c\right)}^{2}\cdot \sigma_{\nabla ▵P_{1}}^{2}+\mu_{\left(i,j,k\right)}^{2}\cdot \sigma_{\nabla ▵\Phi_{1}}^{2}}$$

(26) $$\sigma_{\mathrm{TGF}}=\sqrt{{\left(\beta_{\left(i,j,k\right)}\pm \beta_{\left(a,b,c\right)}\right)}^{2}\cdot \sigma_{\nabla ▵I_{1}}^{2}+\mu_{\left(a,b,c\right)}^{2}\cdot \sigma_{\nabla ▵P_{1}}^{2}+\mu_{\left(i,j,k\right)}^{2}\cdot \sigma_{\nabla ▵\Phi_{1}}^{2}}$$

(27) $$ratio=\lambda_{\left(i,j,k\right)}/\sigma_{\mathrm{TGF}}$$

The calculation of the theoretical success rate based on the assumptions is briefly introduced here. The ambiguity success rate is also known as the reliability of AR. When the success rate is sufficiently close to 1, the uncertainty of the integer AR can be neglected. The distribution of float ambiguities can be regard as a biased Gaussian normal distribution in units of cycle [35], $\hat{a}∼N\left(a+bias,\sigma \right)$. Where, *a* is the true integer ambiguity; *σ* is the part of standard deviation of the float ambiguities caused by the code and phase noise: $\sigma =\sqrt{\left(\sigma_{\nabla ▵N}^{2}-bias^{2}\right)}$. Where, $\sigma_{\nabla ▵N}$ is the standard deviation of the GF float ambiguities, which can be calculated by applying the variance-covariance propagation law to Equation (10), (13) or (16). Biases in the float ambiguities could be generated by outliers in the code data, cycle slips in the phase data, multi-path or the presence of unaccounted atmospheric delays, while in this study, bias is mainly considered as the effect of the ionosphere. Thus the probability of fixing $\hat{a}$ into *a* equals fixing the bias into zero. Then, the GF ambiguity success rate is given as follows.

(28) $$P\left(-0.5<x<0.5\right)=\int_{-0.5}^{0.5}\frac{1}{\sigma \sqrt{2\pi }}\text{exp}\left(-\frac{{\left(x-bias\right)}^{2}}{2\sigma^{2}}\right)\text{dx}$$

**Figure 2:**
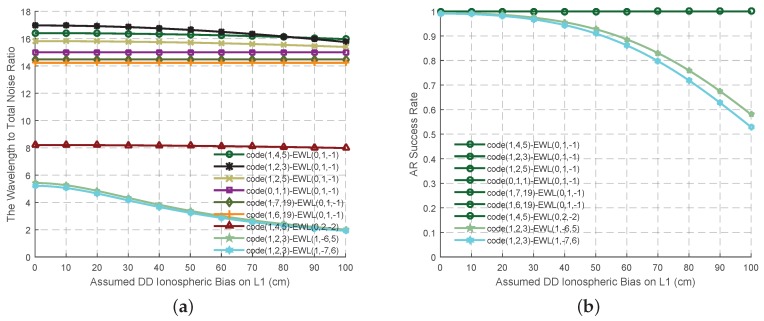
The variation trend of the wavelength to total noise ratio and the AR success rates of the signal pairs in Table 1 for GF TCAR. (**a**) The wavelength to total noise ratio; (**b**) AR success rates.

With regards to the former four criteria, the next mission is to determine the most useful signal pair out of the three fundamental carrier phases and the three original codes for the GF TCAR purpose. The combined signal pairs containing the combined code signal and EWL phase signal are firstly selected as shown in Table 1. Only four EWL phase signals meet the criteria discussed before, while only two of them, EWL(0, 1, −1) and EWL(1, −6, 5) are linear independent. The wavelength to total noise ratio and the AR success rate of all these signal pairs are analyzed in Figure 2 updating with the ionospheric bias. Compared with EWL(0, 1, −1), the combined signal EWL(1, −6, 5) has a comparable success rate when the ionospheric bias is not high, although the ISFs and the measurement noise are bigger and the wavelength to total noise ratio is smaller. However, the success rate of EWL(1, −6, 5) will decrease significantly with the increase of the ionospheric bias, while EWL(0, 1, −1) still maintains a high success rate. Thus in this study, EWL(0, 1, −1) is selected as the best EWL combined phase signal, which can nearly work well with all the combined code signals. With regards to the ionosphere free aspect and the 100% AR success rate, the signal pair code(0, 1, 1)-EWL(0, 1, −1) is selected as the optimal EWL signal pair as most of the existed papers did, although the measurement noise may be larger and the wavelength to total noise ratio is smaller than some pairs working with some other combined code measurement.

**Figure 3:**
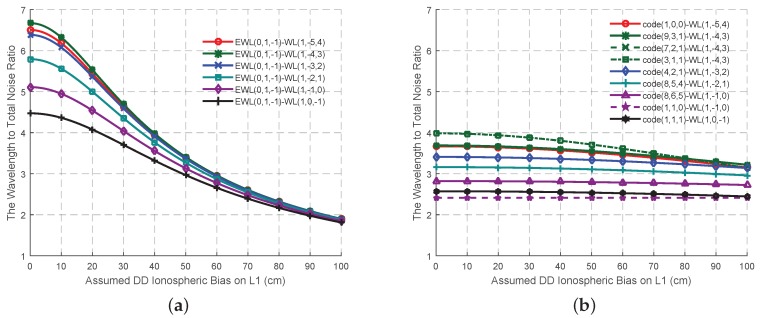
The variation trend of the wavelength to total noise ratio of the WL signal pairs for GF TCAR. (**a**) Signal pairs in Table 2; (**b**) Signal pairs in Table 3.

**Figure 4:**
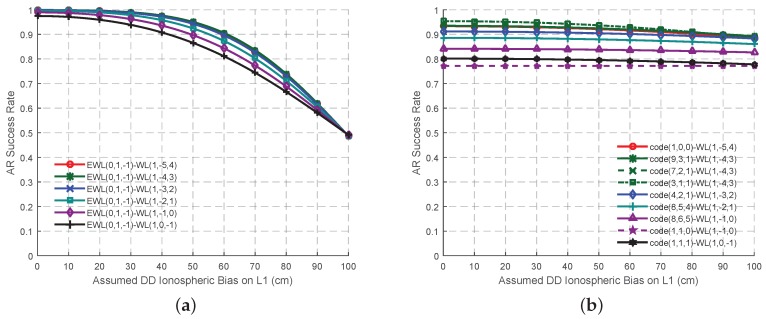
The variation trend of the AR success rates of the WL signal pairs for GF TCAR. (**a**) Signal pairs in Table 2; (**b**) Signal pairs in Table 3.

On the basis of the best EWL combined phase signal and the four criteria, six possible WL signals are selected, as shown in Table 2. When the effect of the ionospheric bias is not as high as the measurement noise, the wavelength to total noise ratios of these signals (Figure 3a) are all larger than 4, while the AR success rates of these six signal pairs (Figure 4a) are high and comparable. At this circumstance, the signal pair EWL(0, 1, −1)-WL(1, −4, 3) is supposed to provide the most reliable ambiguities on account of its wavelength to total noise ratio. With the increase of the ionospheric bias, all the wavelength to total noise ratio will decline to less than 2, while all the AR success rate will be no larger than 0.5. Thus at this stage, it is hard to say which signal pair should be selected as the optimal. However this might be due to the incorrect assumptions of the effect of the error sources, especially ionospheric biases [16]. Two methods are proposed here to solve this problem. One is to be refined with the real data, which will be conducted in the next section. The other adopts combined code signals to work with the WL signals when the ionospheric bias becomes serious, as shown in Table 3. Compared to the signal pairs in Table 2, although these in Table 3 are a little nosier, which lead to a lower wavelength to total noise ratio at the beginning (Figure 3b), they have a stronger ability to resist the effect of the ionospheric bias as the ISFs are smaller. Thus, the wavelength to total noise ratio and the AR success rate do not decline much with the growth of the ionospheric bias, as shown in Figure 4b. The signal pairs involved with WL(1, −5, 4), WL(1, −4, 3) and WL(1, −3, 2) can maintain the success rate is larger than 0.9 no matter how serious the ionospheric bias is, which might be acceptable for AR when using smoothing techniques.

**Figure 5:**
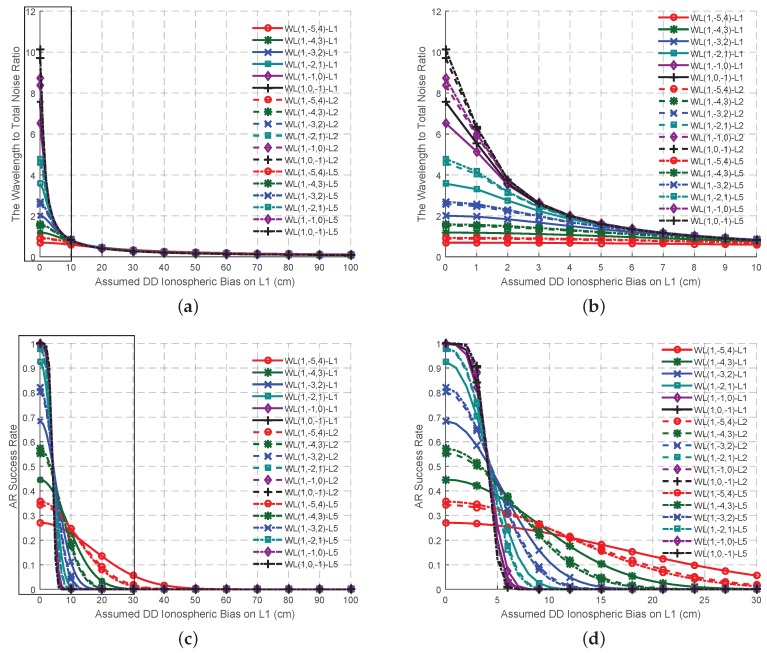
The variation trend of the wavelength to total noise ratio and the AR success rates of the signal pairs of GF TCAR in Table 4 updates with the DD ionospheric bias on L1. (**a**) The wavelength to total noise ratio; (**c**) AR success rate; (**b**,**d**) are the amplification of the rectangle in (**a**,**c**) respectively.

Besides working with EWL ambiguity corrected signals and combined code signals, for WL signals, another considerable aspect is to work with the fundamental signals, whose ambiguities are of the interest when high precise positioning is needed. Table 4 shows the ISFs and the measurement noise of the WL signals working with the fundamental signals, while the wavelength to total noise ratio and the AR success rate are shown in Figure 5a,c and partly amplified in Figure 5b,d updating with the DD ionospheric bias on L1. When the affect level of the DD ionospheric bias is less than approximate 2 cm, the signal pairs, WL(1, −1, 0) and WL(1, 0, −1), are clearly at a priori stage to be adopted as the optimal, on account of the high wavelength to total noise ratio and AR success rate. However it is not easy to decide the ambiguity of which fundamental signal should be solved first as both the wavelength to total noise ratio and the success rate are similar for a certain WL signal. When the effect of the DD ionospheric bias increases, both the wavelength to total noise ratio and the AR success rates of all the signal pairs decline significantly into a value near zero, making it difficult to decide the optimal signal pair for AR. The bad AR performances of WL-single signal pairs might be because of the high ISFs of these signal pairs, as well as the incorrect assumptions problem, which will be tested in the next section in order to select the optimal WL phase signal and the corresponding signal pair. Here we also find that there is a strong correlation (0.9337), between the wavelength to total noise ratio and AR success rate. In order to obtain a 100% success rate, the approximate wavelength to total noise ratio should be larger than 8.

#### 2.2.2. Theoretical Selection of Combined Signals for GB TCAR

**Figure 6:**
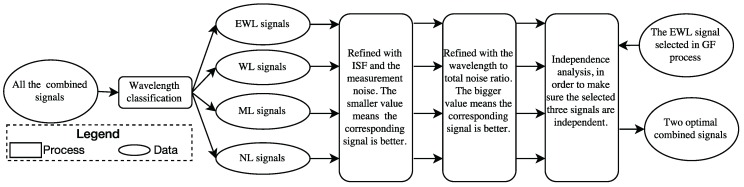
The procedure of selecting the optimal signals for GB TCAR.

As the first step of GB TCAR adopts a GF process, whose optimal EWL phase signal has been theoretically selected, the main aim of this subsection is to determine two extra combined signals, which should be linearly independent from EWL(0, 1, −1). These combined signals can be chosen from EWL, WL, middle-lane (ML, 0.19 m ≤λ< 0.75 m) or narrow-lane (NL, λ<0.19 m) signals, considering the following three criteria, wavelength, ISF and the measurement noise, and the wavelength to total noise ratio. The wavelength is adopted to allocate the classification of the combined signals. The ISF and measurement noise ($\sigma_{\text{GB}}$) will be collaboratively considered in order to select the optimal combined signals with the updating of the frequency dependent error sources, mainly ionospheric biases. On the basis of PNF and the assumption for the DD L1 carrier phase noise, the measurement noise can be calculated with Equation (29). Different from the GF method, GB TCAR cannot eliminate either the frequency dependent error sources or the frequency independent ones, including the satellite orbit error and the tropospheric bias. In this study, the tropospheric bias is assumed to be linearly increased from 0 to 50 cm with the increase of the baseline length, while the orbit error is from 1 to 8 cm [13]. The wavelength to total noise ratio, which can be calculated with Equations (30) and (31), is adopted in order to consider all the error sources in the determination of the optimal combined signals for GB TCAR. With regards to the correlation between the wavelength to total noise ratio and the AR success rate, the larger of the wavelength to total noise ratio is, the higher of the success rate will be. Therefore the optimal combined signals should have the largest wavelength to total noise ratio. Figure 6 summarizes the procedure of selecting the optimal signals for GB TCAR using these criteria.

(29) $$\sigma_{\text{GB}}=\mu_{\left(i,j,k\right)}\cdot \sigma_{\nabla ▵\Phi_{1}}/\lambda_{\left(i,j,k\right)}$$

(30) $$\sigma_{\text{TGB}}=\sqrt{\sigma_{\nabla ▵\delta_{\text{orb}}}^{2}+\sigma_{\nabla ▵\delta_{\text{trop}}}^{2}+\beta_{\left(i,j,k\right)}^{2}\cdot \sigma_{\nabla ▵I_{1}}^{2}+\mu_{\left(i,j,k\right)}^{2}\cdot \sigma_{\nabla ▵\Phi_{1}}^{2}}$$

(31) $$ratio_{\text{GB}}=\lambda_{\left(i,j,k\right)}/\sigma_{\text{TGB}}$$

**Figure 7:**
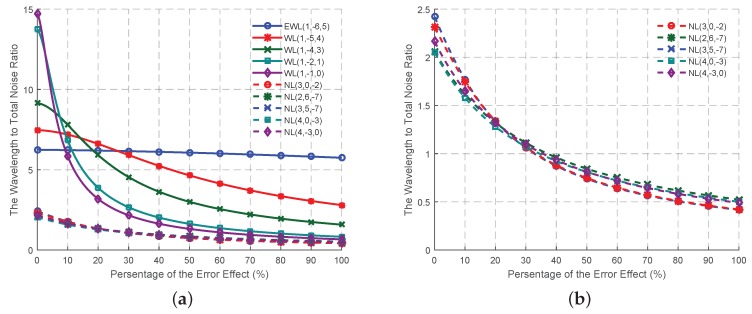
The variation trend of the wavelength to total noise ratio of the selected combined signals in Table 5 updates with the effect percentage of the total noise level from 0 to 100. (**a**) The EWL, WL and NL combined signals; (**b**) The NL combined signals only.

With regards to the former three criteria, ten combined signals, including five EWL/WL signals and five NL signals, are selected, as shown in Table 5. It is impossible to find a combined signal with the smallest ISF, as well as the smallest measurement noise at the same time. However, obviously the wavelength to total noise ratios of the five EWL/WL signals are always larger than those of the NL signals, as shown in Figure 7. We can select only one EWL/WL signal, since none will be independent from the EWL(0, 1, −1) once any of those five signals is chosen. WL(1, −1, 0) is the best signal when the affect of the ionospheric bias, the tropospheric bias and the orbit error are weak. With the increase of the affect of these error sources, each of the five EWL/WL signals has its own period with the best AR performance, while EWL(1, −6, 5) can maintain its performance. The wavelength to total noise ratios of the NL signals are quite similar to each other. NL(3, 0, −2) is the best until the percentage of the error affect exceeds 25. After that, NL(3, 5, −7) performs a little better than any of the others. However, it should be noted that the differences of the wavelength to total noise ratio of all the NL signals are not so obvious, that any one of these five NL signals can have the best performance when using the real data.

## 3. Empirical Experiment and Result Analysis

### 3.1. Basic Information of the Data and the Process Strategy

In order to test if the former assumptions, theories and the selected combined signals are reasonable or not, the signals will be tested using the observations only from the triple-frequency GPS satellites in this section. As the number of triple-frequency GPS satellites is currently not always enough for AR, one hour duration data starting from 6:30 a.m. on 9 April 2016 was collected with the help of mission-planning. During the observation slot, there were six triple-frequency satellites, whose elevation angles to any of the observation stations are over 10°, which is considered to help restrict the influence of multipath. The observations are obtained from nine stations, which are all located in southwest Australia. Stations CUT0 and CUT2 belong to the shared GNSS CORS data of Curtin University, while the other stations are from the Geoscience Australia GNSS anonymous FTP archive. All of the data were obtained using Trimble NetR9 receivers in static mode, with an interval of 30 s and processed in static mode as well. In order to show the changes of the AR performance of different combined signals, eleven baselines with different and reasonable lengths were selected. Table 6 summarizes the basic information of these stations and the data, while the baselines are listed in Table 7. It should be noted that the signals and the data loss rate are only considered with regards to the piece of data adopted in this experiment.

**Figure 8:**
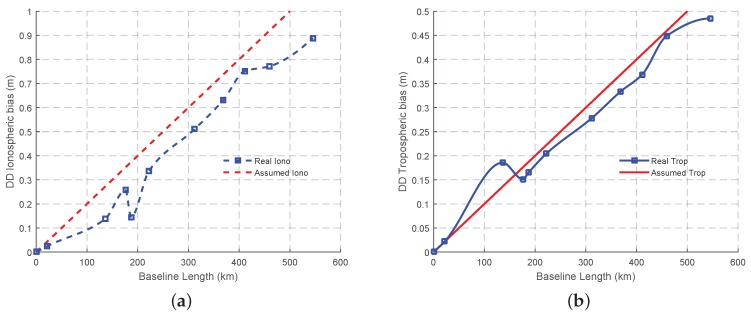
The variation trend of DD ionospheric bias and DD tropospheric bias updates with the baseline length. (**a**) The ionospheric bias; (**b**) The tropospheric bias.

The levels of the effects of the main error sources are analyzed now, including the measurement noise, the ionospheric bias and the tropospheric bias. A zero baseline test is conducted to obtain the measurement noise level of both the carrier phase and the code. Through DD the observations of the zero baseline, the measurement noise of the code and the carrier phase are 0.3 m and 0.002 m respectively. The ionospheric bias of the the original L1 signal is estimated using a Klobuchar model [36], while the tropospheric bias is modeled using a simple method described in [37]. The mean of the DD ionospheric bias and tropospheric bias initially calculated for each baseline, prior to the interpolation method is adopted to obtain the changes of both the ionospheric bias and the tropospheric bias with the increase of the baseline length, as shown in Figure 8. Basically, both DD ionospheric bias and tropospheric bias are approximately linearly increased with fluctuations, which is similar as assumed. Compared to the real value, the assumed value is contiguous. Overall, the assumed observation condition is reasonable, and the real data in this study can be used to test our selected combined signals based on our assumptions. It should be noted that except testing the rationality of the assumed value, the estimated ionospheric and tropospheric parameters will not be used in the process of fixing ambiguities, for the reason that the aim of our research is to identify the optimal signals for different lengths of baselines. One of the good qualities of the optimal signals is the ability to mitigate the effect of the ionospheric bias and the tropospheric bias. If these estimated parameters are adopted to assist AR, the highest AR success rate may be obtained from the combined signals with low capability to mitigate the biases, which will have an adverse impact on the selection of the combined signals. However, the estimation accuracy of both the ionospheric bias and the tropospheric bias can be improved with the help of IGS products or water vapor radiometer, which has been already studied by some research [9,10,38,39,40]. The data processing strategy and the estimated parameters for both GF and GB mode are summarized in Table 8.

### 3.2. Experiment of the Combined Signal Pairs in GF TCAR

**Figure 9:**
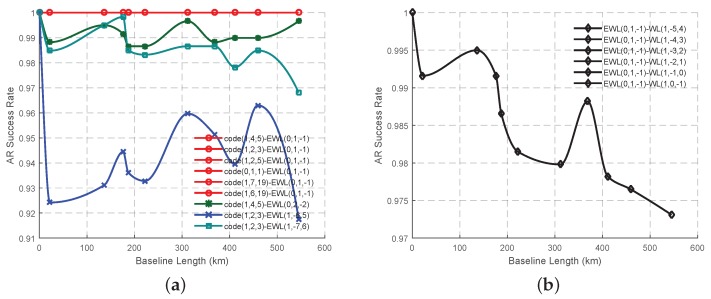
The variation trend of AR success rate in GF TCAR. (**a**) The signal pairs with combined code and EWL carrier phase; (**b**) The signal pairs with ambiguity corrected EWL carrier phase signal and WL carrier phase signal.

**Figure 10:**
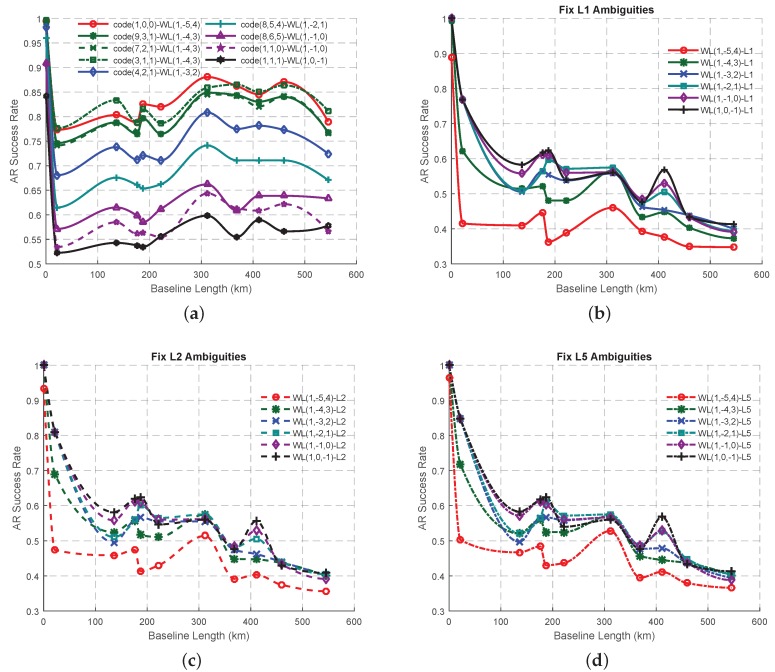
The variation trend of AR success rate in GF TCAR. (**a**) The signal pair with combined code and WL carrier phase; (**b**–**d**) The signal pairs with ambiguity corrected WL carrier phase signal and fundamental signals L1, L2 and L5 respectively.

All the selected combined signal pairs for the GF TCAR will be analyzed in this section using real triple-frequency GPS data in respect of the success rate. In order to assess the effects of the different signal pairs on AR, the success rates will be calculated based on the theory of statistics without using any atmospheric corrections or smoothing techniques. The observations of the eleven baselines are initially used to calculate the success rates of all the signal pairs, after which the Piecewise Cubic Hermite Interpolating Polynomial (PCHIP) interpolation method will be used to obtain the possible changes of the success rate with the increase of the baseline length. The reason why this study adopts PCHIP interpolation method is that the generated lines are easier to see the fluctuations than those from the linear interpolation and the interpolated values are more reasonable than those from higher order non-linear interpolation such as Lagrange, which usually provides too big fluctuations. The success rates of all the combined signal pairs involving EWL(0, 1, −1) (Figure 9a) are the same as expected (Figure 2b), which is 1. Therefore, there will be no EWL signal pairs which can have a better performance than the traditional signal pair, code(0, 1, 1)-EWL(0, 1, −1). The same conclusion can also been drawn with the WL signal pairs (Figure 9b), where all the signal pairs have the same success rate regardless of the baseline length, although the success rate declines with fluctuations. Figure 10a shows the success rates of the signal pairs with both WL carrier phase and combined code start to increase a little when the baseline length becomes longer than around 20 km, after a sharp decline when the baseline length was between 0 and 20 km. This proves that these signal pairs have the ability to restrict the effect of the ionospheric bias, although the success rate still cannot be comparable to those using EWL ambiguity corrected signal pairs. Figure 10b shows that the success rates of all the WL-single signal pairs are declining with the increase of baseline length, while the success rates of the signal pairs with WL(1, 0, −1) or WL(1, −2, 1) have the capability to perform better than the traditional signal pair. Compared to the traditional signal pair, WL(1, 0, −1)-single(1, 0, 0) performs slightly better with an improvement of the AR success rate by about 3% at maximum when the baseline length is less than 200 km or over 350 km, while WL(1, −2, 1)-single(1, 0, 0) can have the best performance when the baseline length is between 200 and 300 km. Comparing Figure 10b–d, it is not easy to decide which fundamental signal should be adopted at a prior stage since the AR success rate curves involving in the same WL signals show the same trend and magnitude when working with different fundamental signals. Comparing Figure 4a,b, Figure 5a, Figure 9b and Figure 10a,b, it can been seen that the assumed success rates using pure carrier phase combined signal pairs will be lower than those using the real data, while the success rates using signal pairs involving both carrier phase and code will be higher than those using real data.

### 3.3. Experiment of the Combined Signals in GB TCAR

The theoretically selected combined signals for GB TCAR will be analyzed in this section based on two criteria, the AR success rate and the reliability ratio. The AR success rate of GB TCAR in this study is calculated by the percentage of (*R*), whose value is larger than a validation threshold ($R_{\text{thres}}$), which is set to 2. *R* is the ratio of the weighted sum of the squared residuals by the second best solutions (${\overset{ˇ}{N}}_{2}$) to ones by the best solutions ($\overset{ˇ}{N}$), as shown in Equation (32) where $\hat{N}$ is the float ambiguity and $Q_{N}^{-1}$ is the corresponding variance-covariance matrix. Although some researchers argue that this calculation method for AR success rate might lead to unnecessary rejections [41], it was still adopted by a number of papers and research software [42,43,44] for convenience, as well as for a reason that a higher *R* value usually means more reliable AR. Therefore, the *R* value will be compared epoch by epoch when the combined signals share the same success rate. The subtraction of the percentage of the epochs, in which the *R* values obtained by one combined signal (signal A) are larger than those obtained by another combined signal (signal B), from the percentage of the epochs, where *R* values of signal B are larger than those of signal A, is called the reliability ratio of A to B. If the reliability ratio is positive, this means the percentage of epochs whose ambiguities can be more reliable using signal A compared to signal B.

(32) $$R=\frac{{\left({\overset{ˇ}{N}}_{2}-\hat{N}\right)}^{T}Q_{N}^{-1}\left({\overset{ˇ}{N}}_{2}-\hat{N}\right)}{{\left(\overset{ˇ}{N}-\hat{N}\right)}^{T}Q_{N}^{-1}\left(\overset{ˇ}{N}-\hat{N}\right)}>R_{\text{thres}}$$

**Figure 11:**
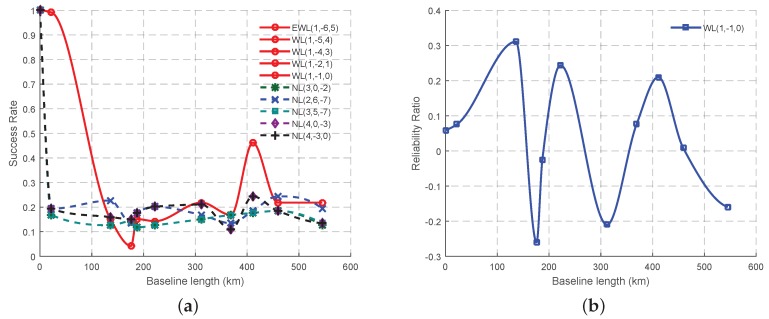
The variation trend of AR success rate in GB TCAR. (**a**) The success rates of all the selected combined signals; (**b**) The reliable ratio of WL(1, −1, 0) compared to EWL(1, −6, 5).

The AR success rate of the eleven baselines are initially calculated to prepare the data which is used to demonstrate the changes with the increase of the baseline length using the PCHIP interpolation method. Figure 11a shows that the EWL and WL signals share the same AR success rate along the increase of the baseline length. Thus the reliability ratio is considered among all the five EWL and WL signals, with a result of the selection of the second optimal EWL and WL signal, WL(1, −1, 0), as shown in Figure 11b. Although, Figure 11b shows a randomness of the reliability ratio, it can be argued that WL(1, −1, 0) can provide more reliable ambiguities than EWL(1, −6, 5) especially in the case that the baseline length is within 100 km from the statistical point of view. Compared to the success rates of the EWL and WL signals, the NL signals will not have a better performance when the baseline length is within 100 km. For the baselines whose lengths are longer than 100 km, it is hard to say which signal possesses the best performance, as all the curves show obvious random volatility. However, among all the NL signals, the traditional NL(4, 0, −3) has the best performance when the baseline length is between 200 and 400 km, as well as NL(4, −3, 0), while NL(2, 6, −7) can be the most outstanding NL signal for the baseline length around 100 km, with a maximum larger value of 8%.

## 4. Conclusions

In this paper, we proposed a theoretical and empirical combined method to select the optimal combined signals for GF TCAR and GB TCAR separately. For GF TCAR, four criteria, including wavelength, the sum of ISFs and the measurement noise, wavelength to total noise ratio and the theoretical success rate, were considered to select the possible optimal combined signal pairs. The theoretically selected combined signal pairs were refined with the real triple-frequency GPS data by considering the AR success rates based on the theory of statistics. For GB TCAR, the possible combined signals were first theoretically selected with regards to three criteria, wavelength, ISF and the measurement noise, and the wavelength to total noise ratio. These signals were refined using the same data sample as GF with regards to the AR success rate and the reliability ratio. The main conclusions and the future research aspects are listed as follows.

1. In GF TCAR, there are no code with EWL signal pair and EWL with WL signal pair which can perform better than the corresponding traditional signal pairs. However, for the WL with single signal pairs, ones working with WL(1, 0, −1) can provide higher success rates by a maximum number of 8% for the baselines whose length are shorter than 200 km or over 400 km, while for other lengths of baselines, ones with WL(1, −2, 1) can perform better compared to the traditional signal pairs, which work with WL(1, −1, 0). The ambiguity corrected EWL carrier phase signals perform better than any combined code signals in AR when fixing ambiguities of WL signals. When working with the EWL corrected signal, all the WL signal pairs proposed by this paper can have the same success rate regardless of the baseline length.
2. In GB TCAR, the WL(1, −1, 0) signal selected in this paper has higher probability to provide more reliable ambiguities especially when the baseline length is short, compared to the traditional EWL(1, −6, 5) signal. The combined signal NL(3, 5, −7) performs more stable than the traditional NL(4, 0, −3), while NL(4, −3, 0) always shares the same success rate with NL(4, 0, −3).
3. In GF TCAR, although the results obtained by the theoretical section meet the results using the real data, the assumed success rates using pure carrier phase combined signal pairs will be lower than those using the real data, while the success rates using signal pairs involving both carrier phase and code will be higher than those using real data. To the best of our knowledge, this might be because of the slightly incorrect assumptions of the effect of the error sources and the imprecision of the mathematic model used in the theoretical analysis. However, the real reason needs further research.
4. In GB TCAR, although the proposed theoretical method can be used to analyze the relationship between the signals and the noise level, there was not showing a strong correlation between the wavelength to total noise ratio using the theoretical model and the success rate using the real data, which means there might be still some unselected signals with a good success rate when only the wavelength to total noise ratio was considered. Therefore, future improvement could be on developing a theoretical method to demonstrate the GB success rate directly in order to discover all the signals with high performance.

## Figures and Tables

**Table 1:** The wavelength, ISFs and the measurement noise of the EWL signals working with combined code signals in GF TCAR.

Signal Pairs	$\lambda_{\left(i,j,k\right)}$ (m)	ISFs	$\sigma_{\text{GF}}$ (Cycle)
code(1, 4, 5)-EWL(0, 1, −1)	5.8610	−0.0844	0.0610
code(1, 2, 3)-EWL(0, 1, −1)	5.8610	−0.1381	0.0589
code(1, 2, 5)-EWL(0, 1, −1)	5.8610	−0.0876	0.0632
code(0, 1, 1)-EWL(0, 1, −1)	5.8610	0	0.0667
code(1, 7, 19)-EWL(0, 1, −1)	5.8610	−0.0024	0.0690
code(1, 6, 19)-EWL(0, 1, −1)	5.8610	0.0003	0.0703
code(1, 4, 5)-EWL(0, 2, −2)	2.9305	−0.0844	0.1219
code(1, 2, 3)-EWL(1, −6, 5)	3.2561	1.5060	0.1845
code(1, 2, 3)-EWL(1, −7, 6)	7.3263	3.5612	0.1918

**Table 2:** The wavelength, ISFs and the measurement noise of the EWL-WL signal pairs for GF TCAR.

Signal Pairs	$\lambda_{\left(i,j,k\right)}$ (m)	ISFs	$\sigma_{\text{GF}}$ (Cycle)
EWL(0, 1, −1)-WL(1, −5, 4)	2.0932	1.0570	0.1537
EWL(0, 1, −1)-WL(1, −4, 3)	1.5424	0.7788	0.1499
EWL(0, 1, −1)-WL(1, −3, 2)	1.2211	0.6166	0.1566
EWL(0, 1, −1)-WL(1, −2, 1)	1.0105	0.5103	0.1726
EWL(0, 1, −1)-WL(1, −1, 0)	0.8619	0.4352	0.1957
EWL(0, 1, −1)-WL(1, 0, −1)	0.7514	0.3794	0.2236

**Table 3:** The wavelength, ISFs and the measurement noise of the code-WL signal pairs for GF TCAR.

Signal Pairs	$\lambda_{\left(i,j,k\right)}$ (m)	ISFs	$\sigma_{\text{GF}}$ (Cycle)
code(1, 0, 0)-WL(1, −5, 4)	2.0932	0.3384	0.2727
code(9, 3, 1)-WL(1, −4, 3)	1.5424	0.2344	0.2711
code(7, 2, 1)-WL(1, −4, 3)	1.5424	0.2323	0.2720
code(3, 1, 1)-WL(1, −4, 3)	1.5424	0.3025	0.2510
code(4, 2, 1)-WL(1, −3, 2)	1.2211	0.1519	0.2934
code(8, 5, 4)-WL(1, −2, 1)	1.0105	0.1203	0.3164
code(8, 6, 5)-WL(1, −1, 0)	0.8619	0.0815	0.3547
code(1, 1, 0)-WL(1, −1, 0)	0.8619	0	0.4147
code(1, 1, 1)-WL(1, 0, -1)	0.7514	0.0950	0.3890

**Table 4:** The ISFs and the measurement noise of the WL signals working with fundamental signals for GF TCAR.

Signal Pairs	ISFs			$\sigma_{\text{GF}}$ (Cycle)		
	L1	L2	L5	L1	L2	L5
WL(1, −5, 4)	1.6616	2.3085	2.4549	1.4483	1.1286	1.0815
WL(1, −4, 3)	1.9397	2.5867	2.7330	0.8452	0.6586	0.6311
WL(1, −3, 2)	2.1020	2.7489	2.8953	0.4979	0.3879	0.3718
WL(1, −2, 1)	2.2083	2.8552	3.0016	0.2798	0.2180	0.2090
WL(1, −1, 0)	2.2833	2.9303	3.0766	0.1531	0.1193	0.1144
WL(1, 0, −1)	2.3391	2.9861	3.1324	0.1321	0.1030	0.0987

**Table 5:** Possible combined signals for GB TCAR and their wavelength, ISF and the measurement noise.

Combined Signals	$\lambda_{\left(i,j,k\right)}$ (m)	ISF	$\sigma_{\mathrm{GB}}$ (Cycle)
EWL(1, −6, 5)	3.2561	−0.0744	0.1594
WL(1, −5, 4)	2.0932	−0.6616	0.1316
WL(1, −4, 3)	1.5424	−0.9397	0.1042
WL(1, −2, 1)	1.0105	−1.2083	0.0525
WL(1, −1, 0)	0.8619	−1.2833	0.0333
NL(3, 0, −2)	0.1263	0.2136	0.0881
NL(2, 6, −7)	0.1314	0.2252	0.1916
NL(3, 5, −7)	0.1140	0.0256	0.1886
NL(4, 0, −3)	0.1081	−0.0099	0.1205
NL(4, −3, 0)	0.1145	0.0902	0.1217

**Table 6:** Information of the stations and the data.

Stations	Location	Antenna Type	Interval	Signals	Data Loss Rate
CUT0	Perth	TRM59800.00 SCIS	30 s	GPS L1/L2/L5	0
CUT2	Perth	TRM59800.00 SCIS	30 s	GPS L1/L2/L5	0
PERT	Perth	TRM59800.00 NONE	30 s	GPS L1/L2/L5	0
BURA	Burakin	JAVRINGANT_DM SCIS	30 s	GPS L1/L2/L5	0
KELN	Kellerberrin	JAVRINGANT_DM SCIS	30 s	GPS L1/L2/L5	0
MTMA	Mt. Magnet	LEIAR25.R3 LEIT	30 s	GPS L1/L2/L5	0
WAGN	Wagin	LEIAR25.R3 LEIT	30 s	GPS L1/L2/L5	0
KALG	Kalgoorlie	JAVRINGANT_DM SCIS	30 s	GPS L1/L2/L5	0
RAVN	Ravensthorpe	JAVRINGANT_DM SCIS	30 s	GPS L1/L2/L5	0

**Table 7:** The length of baselines.

Stations	Length	Stations	Length	Stations	Length
CUT0-CUT2	0 km	BURA-PERT	187 km	BURA-KALG	411 km
CUT0-PERT	22 km	WAGN-PERT	222 km	MTMA-KALG	459 km
CUT0-BURA	136 km	KELN-RAVN	312 km	CUT0-KALG	545 km
CUT0-KELN	176 km	KELN-KALG	369 km		

**Table 8:** Triple-frequency data processing strategy and estimated parameters.

Item	GF Mode	GB Mode
Observations	DD phase and code	DD phase and code
Combination mode	Combined signal pairs	Combined signals
Float estimation	Difference within signal pairs	LSQ
integer estimation	Round off	Integer LSQ
Weight	Equal weight	Noise value dependent
Ionospheric bias	Mitigated using combined signal pairs	Mitigated using signal pairs
Tropospheric bias				
Success rate	Statistics	Threshold value and reliable ratio
